# Accuracy of tooth‐implant impressions: Comparison of five different techniques

**DOI:** 10.1002/cre2.737

**Published:** 2023-04-12

**Authors:** Amirhossein Fathi, Mansour Rismanchian, Atousa Yazdekhasti, Masih Salamati

**Affiliations:** ^1^ Dental Prosthodontics Department, School of Dentistry, Dental Materials Research Center Isfahan University of Medical Sciences Isfahan Iran; ^2^ Dental Prosthodontics Department, School of Dentistry, Dental Implants Research Center Isfahan University of Medical Sciences Isfahan Iran; ^3^ School of Dentistry Isfahan University of Medical Sciences Isfahan Iran

**Keywords:** dental implants, dental impression technique, dimensional measurement accuracy, tooth

## Abstract

**Purpose:**

To compare the accuracy of five different tooth‐implant impression techniques.

**Materials and Methods:**

In this in vitro, experimental study, an acrylic model containing one bone‐level Straumann dental implant at the site of maxillary first molar and an adjacent second premolar prepared for a porcelain fused to metal restoration was used. Impressions were made from the model using five different one‐step tooth‐implant impression techniques including scanning with an intraoral scanner, occlusal matrix, wax relief, closed‐tray, and open‐tray techniques. Each technique was repeated 15 times. The impressions were poured with dental stone, and the obtained casts were scanned by a laboratory scanner. The scan file of each technique was compared with the scan file of the original acrylic model by Geomagic Design X software. Data were analyzed by one‐way analysis of variance, and Tamhane's post‐hoc test (*α* = 0.05).

**Results:**

For dental implant, intraoral scanning had the highest accuracy (0.1004 mm^2^) followed by open‐tray (0.1914 mm^2^), occlusal matrix (0.2101 mm^2^), closed‐tray (0.2422 mm^2^), and wax relief (0.2585 mm^2^) techniques (*p* < 0.05). For the prepared tooth, wax relief (0.0988 mm^2^) had the highest accuracy followed by occlusal matrix (0.1211 mm^2^), open‐tray (0.1663 mm^2^), closed‐tray (0.1737 mm^2^), and intraoral scanning (0.4903 mm^2^) technique (*p* < 0.05). For both dental implant and prepared tooth, occlusal matrix (0.2431 mm^2^) had the highest accuracy followed by open‐tray (0.2574 mm^2^), wax relief (0.2693 mm^2^), closed‐tray (0.2862 mm^2^), and intraoral scanning (0.3192 mm^2^) technique (*p* > 0.05).

**Conclusion:**

The compared simultaneous tooth‐implant impression techniques had comparable accuracy with no significant difference.

## 1 INTRODUCTION

Dental impression is a negative or positive imprint of the teeth and adjacent structures for use in dentistry. It is used as a permanent record for the fabrication of restorations or dentures (Alikhasi et al., 2012, 2015; Fathi, Atash et al., 2023; Malik et al., 2018). Impression accuracy can significantly affect the accuracy of the gypsum cast, and the accuracy of the final impression sent to a laboratory plays a fundamental role in marginal adaptation and the internal gap of final restoration (Alikhasi et al., 2012; Malik et al., 2018). Thus, a precise impression is a prerequisite for optimal fit of restoration and treatment success (Malik et al., 2018).

Dental implants are a standard well‐accepted modality for replacement of the lost teeth. Long‐term success of implant‐supported restorations depends not only on implant osseointegration after the surgical phase, but also on durability of osseointegration after prosthetic loading (Fathi, Atash et al., 2023). To achieve this goal, the restoration framework must have a passive fit on dental implants. Precise impression making from dental implants is one of the influential factors in achieving passive fit (Alikhasi et al., 2015). Inaccurate impressions or prosthetic fabrication can lead to incomplete seating of restoration, which results in insufficient adhesion, microleakage, tooth hypersensitivity, dental caries, need for endodontic treatment, and gingival inflammation (Di Fiore et al., 2019; Fathi, Hashemi et al., 2023) and inflict delayed pain and discomfort on the patient (Shadmehr et al., 2021). Utilizing digital tools has been lucrative in various fields of dentistry including radiology and prosthodontics. Digital impression systems emerged at the start of the 1980s as Werner Mörmann started to consider how to create one session treatments. The development of digital impression devices with optical reading technologies has therefore begun (Mörmann, 2004, 2006; Mehdizadeh et al., **​** 2023; Rekow, 2006). Comparing digital and traditional impression processes reveals some similarities and differences (Abdel‐Azim et al., 2015; Amin et al., 2017; Christensen, 2008; Rehmann et al., 2013; Su & Sun, 2016; Wöstmann et al., 2005). A higher number of stages in the conventional impression technique raises the risk of further errors (Alikhasi et al., 2017; Chochlidakis et al., 2016; Ender & Mehl, 2015; Revilla‐León et al., 2021; Tomita et al., 2018; Wöstmann et al., 2008). The standardization of the milling stage in the digital impression process, as well as the use of fewer step numbers, reduces the chance of errors and enhances adaptability (Gimenez‐Gonzalez et al., 2017; Lin et al., 2015; Papaspyridakos et al., 2016; Wöstmann et al., 2005). In terms of time and physician choice, digital approaches are preferred more (Lee & Gallucci, 2013; Schepke et al., 2015; Yuzbasioglu et al., 2014).

However, to increase the accuracy of implant impressions and minimize misfits in conventional techniques, such as the open‐tray and closed‐tray techniques have been proposed, which can be adopted depending on the case. For instance, the closed‐tray technique is more suitable for cases with limited edentulous space, or difficult placement of impression coping in the posterior region (Burns et al., 2003; Carr, 1991). Although impression technique is an important parameter in accuracy of impressions, impression material is another important factor determining the precision of the final cast. For instance, in the occlusal matrix technique, apical pressure applied to the impression material results in more accurate recording of dental margins. For the direct dental impression technique, the impression material should have two properties: adequate stiffness to prevent the rotation of impression coping in the impression, and optimal dimensional stability (Alikhasi et al., 2012).

In partial edentulism, some clinical scenarios may necessitate simultaneous impression making from both implant and an adjacent prepared tooth. Simultaneous tooth‐implant impression techniques decrease the possible errors that may occur during adaptation of final restoration of the prepared tooth and dental implant, save time, and prevent impression material waste. Thus, it is imperative to adopt an acceptable impression technique with a suitable impression material in terms of dimensional accuracy and recording of details (Alikhasi et al., 2015). In such conditions, a common strategy is to use impression copings and two‐phase one‐step impression technique. However, this technique does not address the necessary differences in reconstruction of the prepared tooth and dental implant. Thus, several strategies have been proposed to solve this problem: (i) treatment is divided into two phases, and tooth and implant restorations are fabricated separately; (ii) two impression techniques are employed in one‐step such that a custom tray for the prepared tooth is filled with impression material and seated on the respective area, and then impression copings are placed, and a second impression is made, which includes the first tray and impression copings; (iii) tooth and dental implant frameworks are fabricated after making separate impressions from each of them, and are tried‐in; next, a pick‐up impression is made (Chee & Alexander, 1998; Lorenzoni et al., 2000; Rehmann et al., 2013; Wöstmann et al., 2005). Since studies on the accuracy of simultaneous tooth‐implant impression techniques are limited, this study aimed to compare the accuracy of five different tooth‐implant impression techniques.

## 2 MATERIALS AND METHODS

In this in vitro, experimental study, the accuracy of five different one‐step tooth‐implant impression techniques including scanning with an intraoral scanner, occlusal matrix, wax relief, closed‐tray, and open‐tray techniques was evaluated. Each technique was repeated 15 times, and putty‐wash impression material (Speedex) was used in all techniques. Scanning by a laboratory scanner (UP3D; UP360+; Aniwaa) served as the gold standard in this study for the purpose of comparison with other techniques.

The sample size was calculated to be 15 in each group based on previous similar studies (Alikhasi et al., 2012, 2015; Malik et al., 2018).

**Figure 1:**
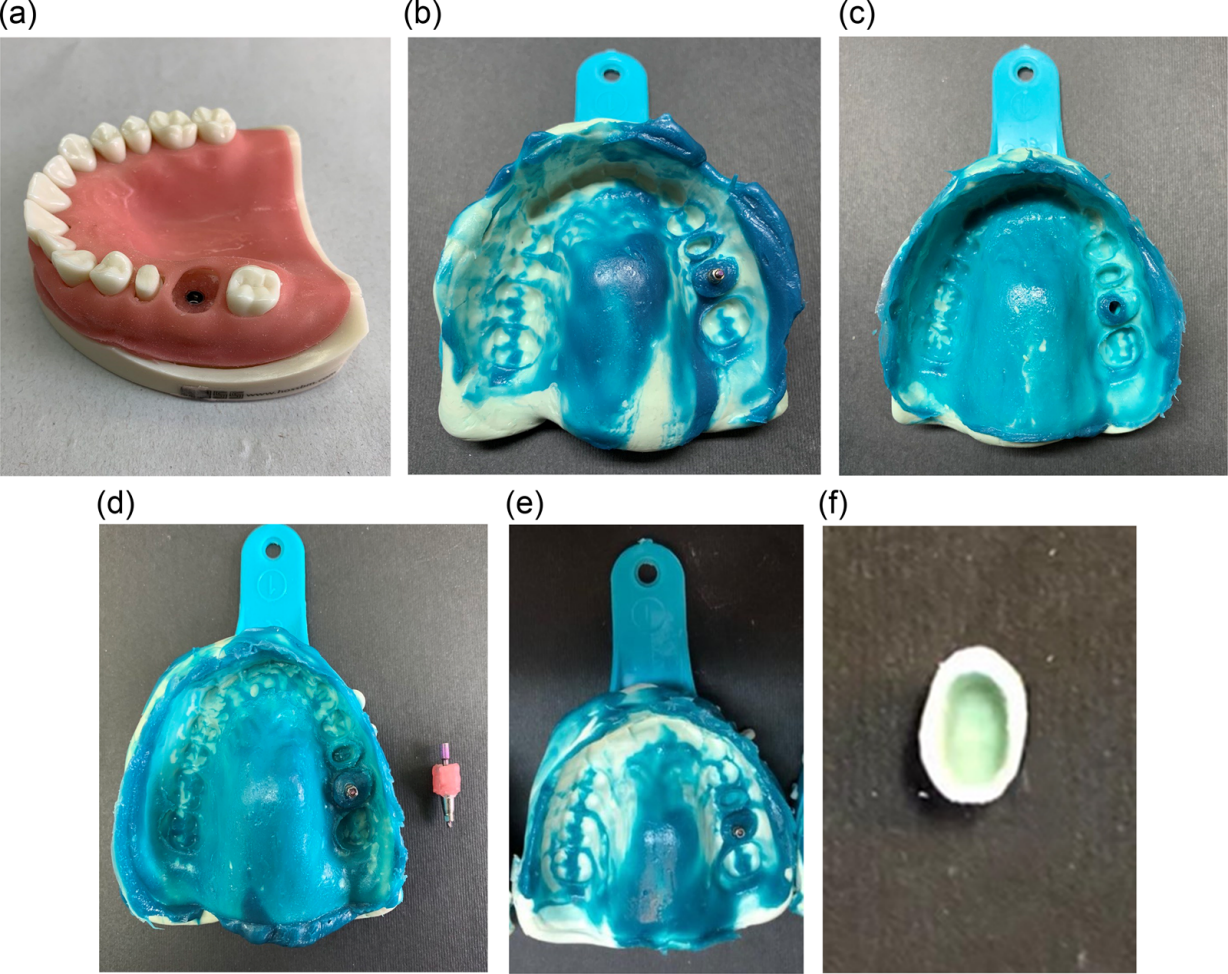
(a) Master model, (b) open tray impression, (c) close tray impression (d) wax relief impression, (e) occlusal matrix impression, (f) occlusal matrix putty.

A reference phantom model of the maxilla with simulated gingiva, soft tissue, and acrylic teeth (HT207U; Hoss) was used in this study, which included one dental implant at the site of maxillary first molar, and an adjacent second premolar prepared for a porcelain fused to metal restoration. To insert dental implants, an implant hole was prepared parallel to the teeth at the site of the maxillary first molar with 4.1 mm diameter and 12 mm height. A bone‐level dental implant (Straumann) was placed in the hole and fixed with autopolymerizing acrylic resin such that the implant surface was at the level of the acrylic surface. Next, impressions were made from the acrylic model with five different techniques as follows (Figure 1):

1. *Scanning with a digital scanner*
  An impression coping was placed on the dental implant, and the acrylic model was scanned with a scanner (Omnicam CEREC). The values were measured and recorded by Sirona Connect version 4.6 software.
2. *Occlusal matrix technique*
  A putty impression was made from the prepared second premolar, and after setting, the putty was removed and trimmed to the size of the prepared tooth margins. Next, an open‐tray impression coping was placed on the dental implant. Wash material was injected into the putty impression made from the prepared tooth, and the impression was placed over the tooth. Wash material was also injected around the implant and coping with the respective syringes. Next, a pick‐up impression was made with a proper‐size prefabricated perforated tray filled with putty and wash material.
3. *Wax relief technique*
  After placing the open‐tray impression coping, it was relieved with two layers of red dental wax. Next, a suitable prefabricated tray was used to make a putty impression from the tooth and implant. After the setting of putty, the wax around the coping was removed. Wash material was injected into the tray, and around the tooth and implant, and an impression was made.
4. *Closed‐tray technique*
  The impression coping of closed‐tray technique was placed, and a putty impression was made. Wash material was injected into the putty impression and around the tooth and implant, and an impression was made.
5. *Simultaneous one‐step tooth‐implant impression technique*
  After the placement of open‐tray impression coping, wash material was injected around the tooth and implant. Putty and wash material were also applied in the tray, and a one‐step impression was obtained from the tooth and implant.

All impressions were poured with type 4 dental stone (Vel‐Mix). The obtained gypsum casts and the acrylic model were scanned by a laboratory scanner, and the data were recorded.

**Figure 2:**
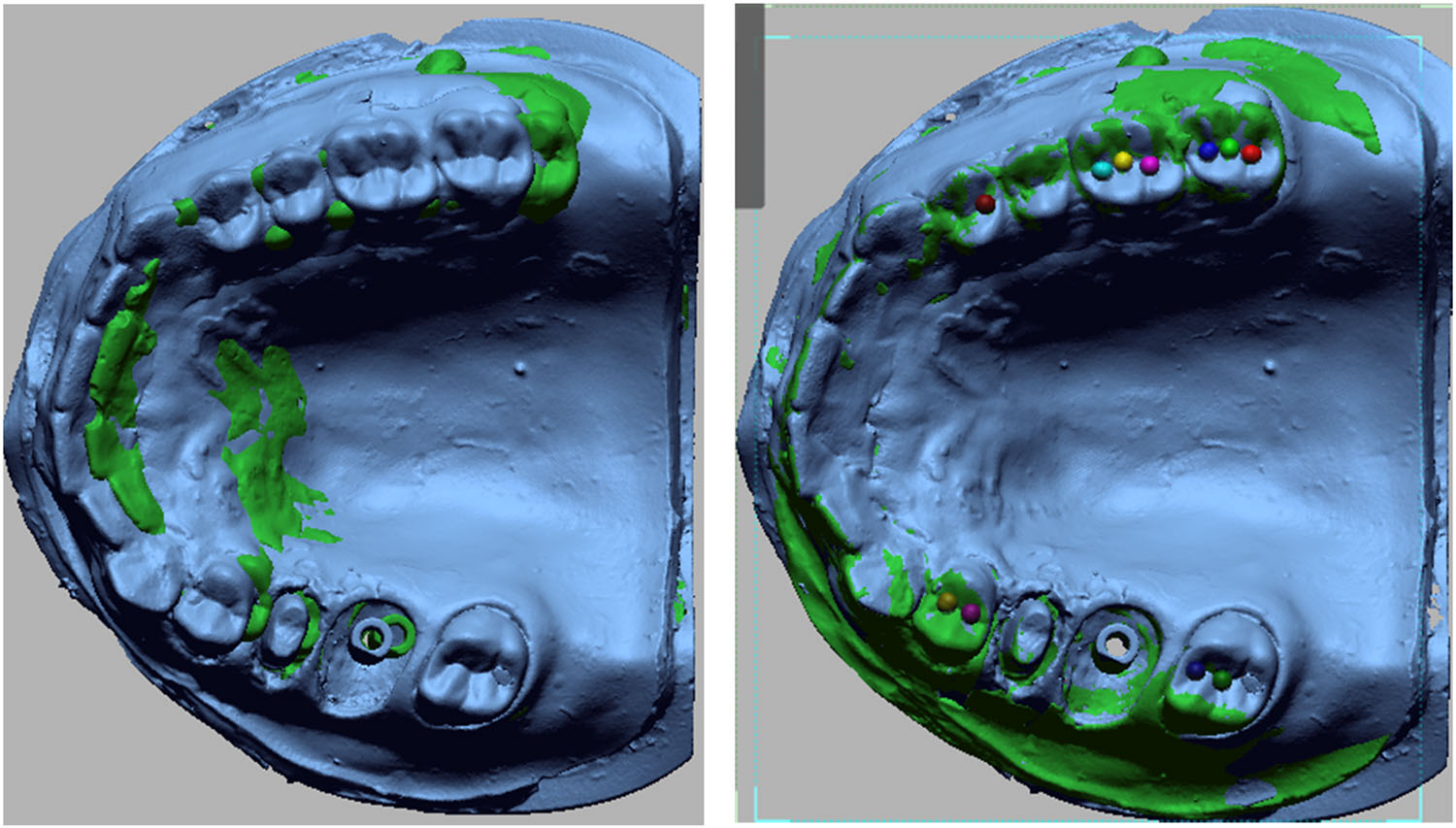
Superimposition of occlusal matrix cast and gold standard files.

**Figure 3:**
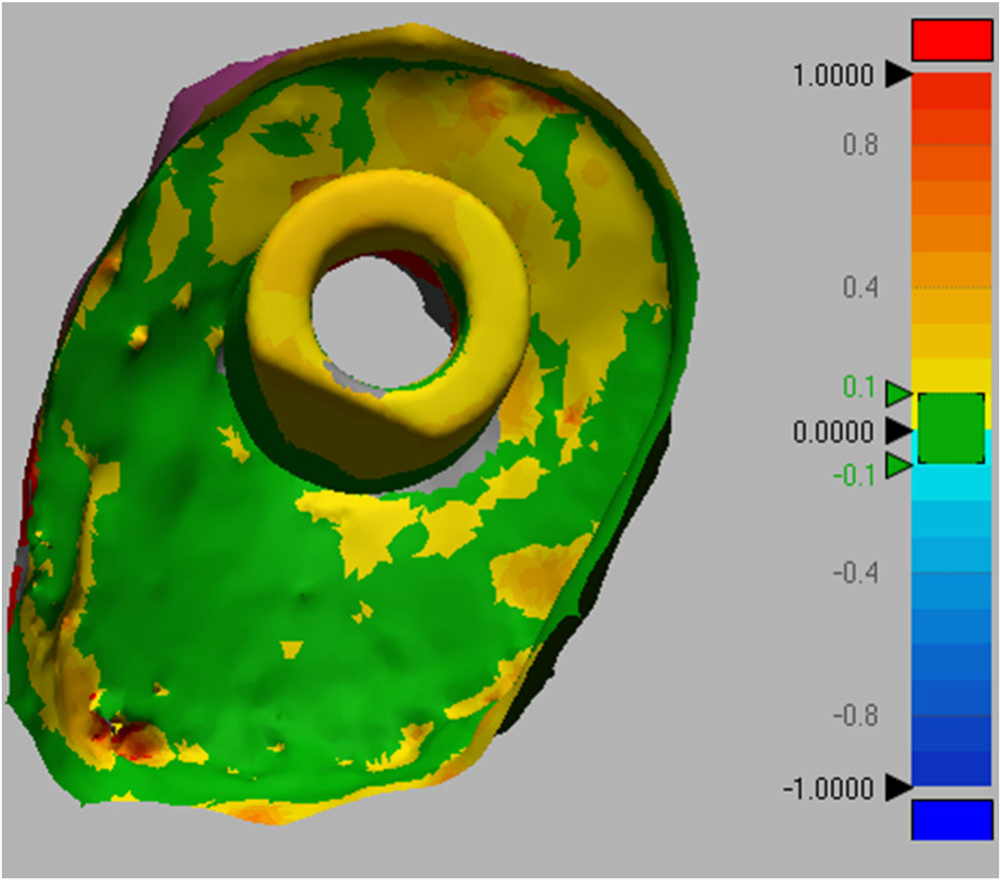
Comparison of occlusal matrix cast and gold standard for dental implant alone.

**Figure 4:**
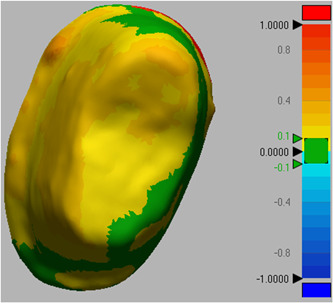
Comparison of occlusal matrix cast and the gold standard for prepared teeth alone.

**Figure 5:**
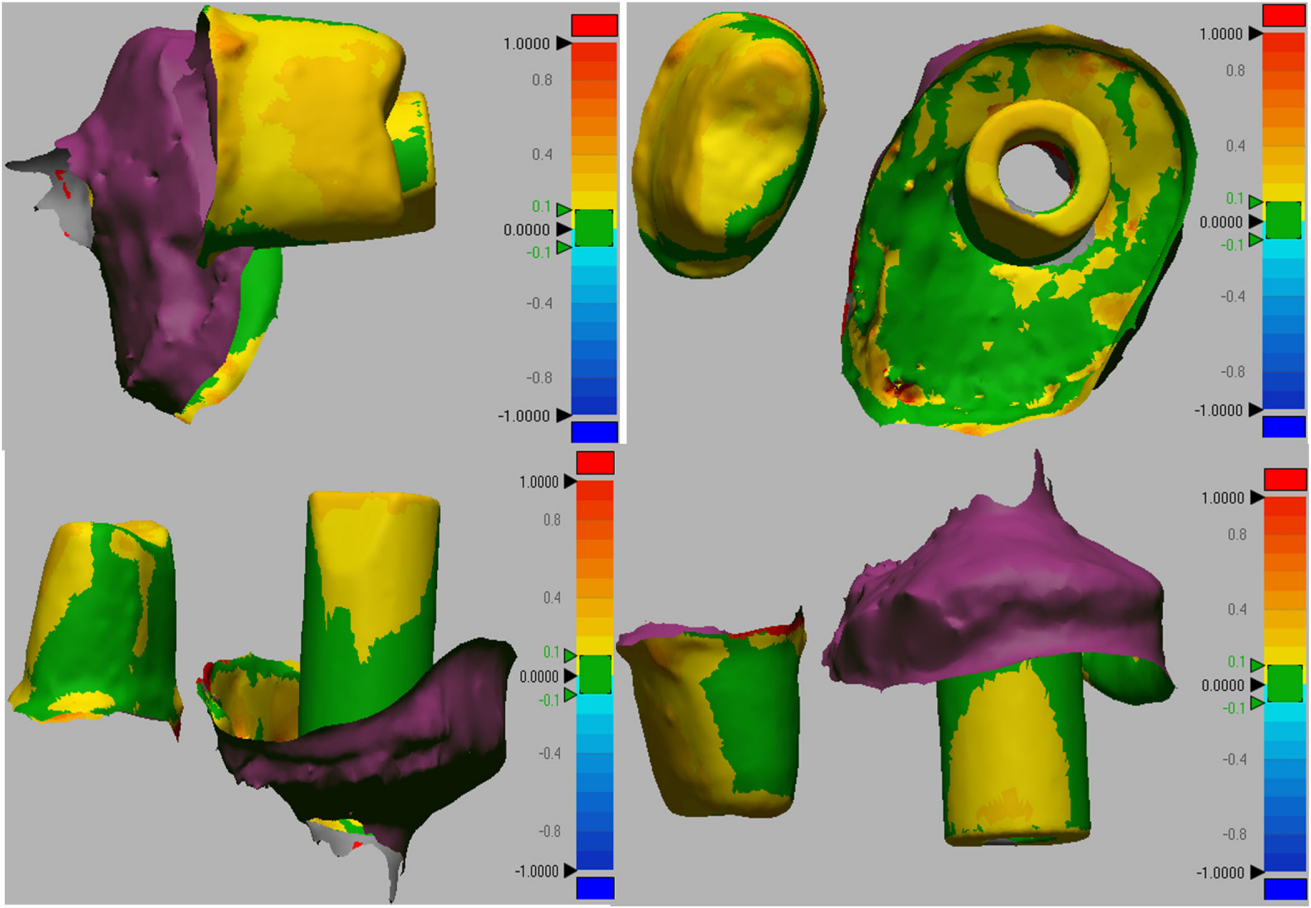
Comparison of occlusal matrix cast and the gold standard for both implant and prepared tooth.

All scan files of the casts in STL format were superimposed on the gold standard file through central and lateral fossae (Figure 2). Next, for better comparison of casts, the implant and prepared tooth areas were cut out of the master cast, and trimmed for more accurate comparison. The casts were compared with the gold standard cast using Geomagic Design X software (Oqton). Accordingly, the mean difference in adaptation of different cast areas with the gold standard was calculated in square millimeters (mm^2^), and reported in three categories of implant alone (Figure 3), prepared tooth alone (Figure 4), and implant plus prepared tooth (Figure 5).

The mean and standard deviation of deviations from the actual value were calculated for each technique. Data were analyzed by SPSS version 22. The normal distribution of data was evaluated by the Shapiro–Wilk test. Comparisons were made by one‐way analysis of variance (ANOVA) followed by the Tamhane's post‐hoc test (for pairwise comparisons) at 0.05 level of significance.

## 3 RESULTS

### 3.1 Simultaneous implants and prepared tooth impressions

Among the simultaneous dental implants and prepared tooth impression techniques, the occlusal matrix had the highest accuracy followed by open‐tray, wax relief, closed‐tray, and intraoral scanning (*p* > 0.05; Table 1).

**Table 1:** Mean difference in the accuracy of different techniques and the gold standard in simultaneous implant and prepared tooth impressions, implant impressions alone, and prepared tooth impressions alone (*n* = 15 for each technique in each category).

Group	Impression technique	Mean	Std. deviation
Dental implant			
	Occlusal matrix	0.2166	0.0614
	Wax relief	0.2328	0.0173
	Open‐tray	0.2118	0.0219
	Closed‐tray	0.2258	0.0369
	Intraoral scanning	0.1050	0.0113
	Total	0.1984	0.0583
Prepared tooth			
	Occlusal matrix	0.1184	0.0203
	Wax relief	0.1087	0.0183
	Open‐tray	0.1282	0.0279
	Closed‐tray	0.1380	0.0319
	Intraoral scanning	0.4843	0.0167
	Total	0.1955	0.1475
Tooth and implant			
	Occlusal matrix	0.2497	0.0409
	Wax relief	0.2621	0.0104
	Open‐tray	0.2562	0.0118
	Closed‐tray	0.2648	0.0385
	Intraoral scanning	0.2784	0.0276
	Total	0.2622	0.0297

### 3.2 Implant impressions

Among dental implant impression techniques, intraoral scanning had the highest accuracy followed by open‐tray, occlusal matrix, closed‐tray, and wax relief techniques (*p* < 0.05; Table 1).

### 3.3 Prepared tooth impressions

Among the prepared tooth impression techniques, wax relief had the highest accuracy followed by occlusal matrix, open‐tray, closed‐tray, and intraoral scanning (*p* < 0.05; Table 1).

The Shapiro–Wilk test showed normal distribution of data. Considering the nonhomogeneity of variances, one‐way ANOVA was applied to compare the accuracy of different techniques within each group, which revealed a significant difference among the five impression techniques for dental implant alone (*p* < 0.001) and also for prepared teeth alone (*p* < 0.001). However, the difference in the accuracy of the five techniques was not significant for simultaneous tooth‐implant category (*p* = 0.094). Table 2 presents pairwise comparisons of the impression techniques for the prepared tooth and implant categories.

**Table 2:** Pairwise comparisons of the accuracy of impression techniques for the prepared teeth and implant categories.

Paired groups compared	*p* Value for prepared tooth alone	*p* Value for implant alone
Occlusal matrix‐wax relief	0.862	0.984
Occlusal matrix‐open tray	0.964	1.000
Occlusal matrix‐closed tray	0.444	1.000
Occlusal matrix‐intraoral scanning	0.000	0.000
Wax relief‐open tray	0.285	0.070
Wax relief‐closed tray	0.053	0.999
Wax relief‐intraoral scanning	0.000	0.000
Open tray‐closed tray	0.992	0.917
Open tray‐intraoral scanning	0.000	0.000
Closed tray‐intraoral scanning	0.000	0.000

## 4 DISCUSSION

Since studies on the accuracy of simultaneous tooth‐implant impression techniques are limited, this study aimed to compare the accuracy of five different tooth‐implant impression techniques. Precision of each impression method is influenced by a number of aspects, including perfect manipulation of impression materials, materials utilized for impression creation, materials used for pouring dental stone, and suitable timing of cast fabrications (Corrente et al., 1995; Daoudi et al., 2001; Hiochwald, 1991; Kupeyan & Lang, 1995; Phillips et al., 1994; Tomita et al., 2018). To evaluate the accuracy of each impression technique in this investigation, a single material (except for intraoral scanning) and all previously indicated criteria were employed. Putty‐wash impression material has qualities that make it a good choice for implant impressions, including exceptional dimensional stability, enhanced accuracy, and replication of fine features (Craig, 1989). Nonetheless, the intraoral scanner and the related software used in this comparison is an accurate and effective intraoral scanner setting (Yuzbasioglu et al., 2014). The results showed that for dental implants alone, intraoral scanning had the highest accuracy followed by open‐tray, occlusal matrix, closed‐tray, and wax relief techniques. For the prepared tooth alone, wax relief had the highest accuracy followed by occlusal matrix, open‐tray, closed‐tray, and intraoral scanning technique. For both dental implants and prepared tooth, occlusal matrix had the highest accuracy followed by open‐tray, wax relief, closed‐tray, and intraoral scanning technique. The difference in the accuracy of the techniques was significant in prepared tooth and implant categories (*p* < 0.05) but not in tooth‐implant category (*p* > 0.05).

Conventional and digital impression techniques each have their own advantages and disadvantages for the fabrication of prosthetic restorations (Christensen, 2008). While most dental clinicians may not be searching for an alternative to conventional impression techniques, digital impression techniques can bring about promising results. A systematic review by Alikhasi et al. (2017) regarding the comparison of digital and conventional impression techniques reported that of the reviewed articles, five studies encouraged dental clinicians to use intraoral scanners for dental implant impressions. Two other studies reported that digital scanning was not reliable, and was not suitable for clinical applications. Since the present results revealed that digital intraoral scanning was the most accurate technique for dental implants, the results of five studies in their systematic review were in line with the present findings regarding the accuracy of dental implant impression techniques. Tomita et al. (2018) reported that the linear measurements made on the reference model by intraoral scanning were smaller than the actual reference values while the measurements made on the scans of casts of conventional impressions were larger than the actual values. Although both techniques were highly accurate and suitable for use in the clinical setting, the authors concluded that intraoral scanning was more accurate than the conventional technique (Tomita et al., 2018). These results were different from the present findings regarding impressions from the prepared tooth but in line with the results regarding dental implant impressions. The reason may be that their study was conducted on a model of the jaw with complete dentition. They used some ceramic balls and measured the distance between them. They also compared three techniques, namely intraoral scanning, alginate impression, and silicone impression techniques. The difference between their results and present findings may be due to the use of alginate impression material.

Revilla‐León et al. (2021), in an in vitro study regarding dental implant impression techniques showed that the conventional technique yielded the most accurate results (minimum three‐dimensional [3D] difference) for implant abutment position. Also, both intraoral scanners yielded reliable results with no significant difference with the conventional method, which was in line with the present findings. A review study by Ahlholm et al. (2018) and Abdel‐Azim et al. (2015) indicated that the accuracy of digital impression technique was comparable to that of conventional technique for the fabrication of crowns and short fixed partial dentures. Their results were different from the present findings because the conventional techniques were more accurate than the digital intraoral scanning method for the prepared tooth in the current study. This difference may be due to different criteria for comparisons in the two studies since Ahlholm et al. (2018) assessed the marginal gap and internal fit two‐dimensionally while we used a 3D superimposition software. Ahlholm et al. (2018) reported that digital impression results were acceptable in terms of clinical fit for the fabrication of implant crowns and implant‐supported fixed partial dentures. Their results regarding the superiority of intraoral digital scanning were in agreement with the present findings. Balamurugan and Manimaran (Balamurugan & Manimaran, 2013) evaluated casts obtained from different dental implant impression techniques and showed that the open‐tray technique was more accurate than the closed‐tray technique for transfer of 3D implant position from the original model to the cast. Their results were in accordance with the present findings. Kumar et al. (2015) demonstrated that the matrix technique was more accurate than the putty‐reline and multiple mix technique for single tooth impressions. The matrix technique used in their study was similar to the occlusal matrix in the present study, and the putty‐reline technique in their study was similar to the wax relief and closed‐tray techniques in the present study. However, Kumar et al. (2015) made impressions from a prepared tooth with a nonperforated tray, which was similar to the tray used for the closed‐tray technique and different from the tray used in the wax relief technique in the present study. The multiple mix technique in their study was similar to open‐tray technique in the current study. Thus, their results were different from the present findings since in the present study the wax relief technique was the most accurate for prepared teeth followed by the occlusal matrix technique. However, due to a very small difference in accuracy of wax relief and occlusal matrix techniques, the present results cannot be considered to be in definite contrast to their findings. Seelbach et al. (2013) measured the internal fit and accessible marginal inaccuracy of fabricated crowns to compare the accuracy of different impression techniques for a prepared tooth. They demonstrated the comparable accuracy of digital and conventional impression techniques. However, their study had an in vitro design, and thus, impressions were made under ideal conditions in absence of blood and saliva contamination or patient's reflexes. Considering the clinical intraoral conditions, it may be stated that digital scanning is a better and safer choice than the conventional technique for routine everyday clinical use. Their results, however, were different from the present findings which may be due to measurement of different indices, and method of comparison of different techniques. Also, they actually fabricated crowns by using the impressions, which may also explain the difference in the results of the two studies. Moreover, they used three types of intraoral scanners in their study versus one scanner in the present study.

According to Ting‐Shu and Jian (2015), although digital impressions have shortcomings such as reproducibility of intraoral impressions, the obtained restorations with the digital technique have an accuracy comparable to that of conventional methods. Although their results were different from the present findings, the positive aspects of digital technique cannot be ignored. Despite the advances in conventional impression materials and their optimal accuracy, the digital impression techniques are clearly superior to the conventional techniques in terms of cost‐effectiveness and preventing material waste.

An important limitation in the implementation of this study was the small number of replicates. It is recommended to conduct this study with a larger number of samples in future studies. Similar in vivo studies and clinical trials are required to compare the accuracy of different tooth‐implant impression techniques in the clinical setting. Also, the accuracy of different impression techniques should be compared using a higher number of prepared teeth and dental implants.

## 5 CONCLUSION

The compared tooth‐implant impression techniques had comparable accuracy with no significant difference. For dental implants alone, digital scanning had the highest and wax relief had the lowest accuracy, while for prepared teeth, wax relief had the highest and digital scanning had the lowest accuracy.

## AUTHOR CONTRIBUTIONS

Amirhossein Fathi conceived and designed the study, collected and analyzed the data, and wrote the initial draft of the manuscript. Mansour Rismanchian contributed to the study design, performed data analysis, and revised the manuscript critically for important intellectual content. Atousa Yazdekhasti contributed to the acquisition and interpretation of data, provided technical support, and revised the manuscript critically for important intellectual content. Masih Salamati contributed to the interpretation of the data, provided scientific and editorial guidance, and revised the manuscript critically for important intellectual content. All authors approved the final version of the manuscript for submission and take responsibility for the integrity of the work as a whole.

## CONFLICT OF INTEREST STATEMENT

The authors declare no conflict of interest.

## Supporting information

Supporting information.

Supporting information.

Supporting information.

## Data Availability

The data that support the findings of this study are available from the corresponding author upon reasonable request.
