# Supplementary material for: Accuracy of tooth‐implant impressions: Comparison of five different techniques
Source: Clin Exp Dent Res. 2023 Apr 12;9(3):526–34. doi: 10.1002/cre2.737 (PMC10280615; doi:10.1002/cre2.737)
Supplement: Supplementary file 2 — Supporting information. [file CRE2-9-526-s003.docx]

Dear Editor:

We would like to express our gratitude to you for your helpful and constructive comments. We really appreciate the points noted by the reviewers and think that by addressing them, our manuscript has improved significantly. We have highlighted the changes made in the text for better tracking. In the following section, we provide a point-by-point reply to the reviewers’ comments:

- **many thanks for the editing of the manuscript and for the answered questions. I am still of the opinion that the number of samples is too few, you could not justify it convincingly.**

Thank you for your comment, we have added now 12 more samples to each group (each group has 15 samples) to improve the quality of our study. The changes in the abstract section, sample size calculation section, results section, and table section are now highlighted. *Pages 1,4,7,15,16,17*
